# Causal effects of sedentary behavior and physical activity on the risk of musculoskeletal disorders: Evidence from Mendelian randomization analysis

**DOI:** 10.1097/MD.0000000000044390

**Published:** 2025-09-19

**Authors:** Xiaoyan Zhang, Yuqiang Li

**Affiliations:** a Key Laboratory of Adolescent Health Assessment and Exercise Intervention of the Ministry of Education, East China Normal University, Shanghai, China; b College of Physical Education and Health, East China Normal University, Shanghai, China.

**Keywords:** body mass index, Mendelian randomization, musculoskeletal disorders, physical activity, sedentary behavior

## Abstract

Previous studies have noted the associations of sedentary behavior and physical activity with several musculoskeletal disorders (MSDs), yet the causality of these relationships remains unclear. In this study, we probed into the causal associations between sedentary behavior, physical activity, and the risk of 13 MSDs through Mendelian randomization (MR) analysis. We obtained summary statistics for leisure screen time (LST) and moderate-to-vigorous intensity physical activity (MVPA) from genome-wide association studies (GWAS) for 2-sample univariable MR analysis. The linkage disequilibrium score regression (LDSC) was used for genetic correlation analysis. Multivariable MR analysis was conducted on LST and MVPA to adjust for one another. Furthermore, we performed mediation analysis to reveal the potential mediating effect of body mass index (BMI). After Benjamini–Hochberg correction, LST was significantly associated with an increased risk of 8 MSDs, including spinal stenosis, spondylolisthesis, low back pain, cervicobrachial syndrome, rotator cuff syndrome, meniscus derangement, lateral epicondylitis, and gout. All these MSDs had positive genetic correlations with LST. There was no causal effect of MVPA on MSDs. However, after adjusting for LST, the association between MVPA and scoliosis became significant; the associations between LST and all 8 MSDs remained after adjusting for MVPA. Two-step mediation analysis found that BMI mediated all the associations between LST and MSDs. Our study provides genetic evidence supporting that sedentary behavior is a causal risk factor for most MSDs independent of physical activity. These findings offer valuable insights for the development of evidence-based prevention strategies and interventions targeting MSDs.

## 1. Introduction

Musculoskeletal disorders (MSDs) constitute a major cause of chronic pain and disability worldwide. They affect individuals across all age groups and lead to impaired quality of life and substantial socioeconomic burden.^[1,2]^ As one of the leading causes of disease burden in the elderly, the burden of MSDs is expected to further increase with the ongoing aging of the global population.^[3,4]^ One of the crucial steps in preventing and managing MSDs is to identify potential protective and risk factors, particularly those that are modifiable.

Leisure sedentary behavior (SB) refers to any waking activities with energy expenditure ≤ 1.5 metabolic equivalents in a reclined or seated position, such as watching TV and computer use.^[5]^ Epidemiological evidence has indicated the associations between SB and a range of adverse health outcomes, including cardiovascular diseases, cancers, and musculoskeletal conditions.^[6–9]^ Prolonged sedentary time has been found to increase the risk of orthopedic diseases, low back pain, rheumatoid arthritis, and other MSDs.^[8,10,11]^

Physical activity (PA) is another factor warranting investigation because of its potential benefits on musculoskeletal health and its intricate interactions with SB.^[12–14]^ While PA is often proposed as a preventive and therapeutic strategy for MSDs,^[15]^ recent evidence indicates that high levels of PA may not fully mitigate the detrimental effects of SB.^[16,17]^ However, traditional observational studies are inherently constrained by residual confounding and reverse causality, which impedes the establishment of causality. Therefore, the causal effects of SB and PA on the risk of MSDs remain to be elucidated.

Mendelian randomization (MR) is an effective analytical method of causal inference at the genetic level. It utilizes genetic variants as instrumental variables (IVs) to ascertain the causal relationship between exposure and outcome.^[18]^ Single nucleotide polymorphisms (SNPs) are randomly allocated at conception, which can effectively minimize bias from confounding factors and reverse causality. Multivariable MR (MVMR) is an extension to MR, and can estimate the direct effect of an exposure on the outcome independent of another exposure. A few studies have applied MR approach to evaluate the associations of SB/PA with several MSDs, mainly focusing on the associations with low back pain, intervertebral disc disorder and arthritis.^[19–21]^ Nevertheless, few studies have systematically investigated the independent causal effects of SB and PA on various MSDs through 2-sample MR and MVMR.

Furthermore, SB and PA are highly correlated with obesity.^[22]^ Meanwhile, recent evidence indicated that obesity-related metabolic changes could contribute to musculoskeletal tissue damage.^[23]^ Body composition has been shown to increase the risk of musculoskeletal chronic pain.^[24–26]^ Therefore, we hypothesized that SB and PA might affect MSDs by influencing body mass index (BMI).

In this study, we conducted MR analysis to examine the causal associations of SB and PA with a wide spectrum of MSDs, and to explore the potential mediating pathway.

## 2. Methods

### 2.1. Study design

**Figure 1.**
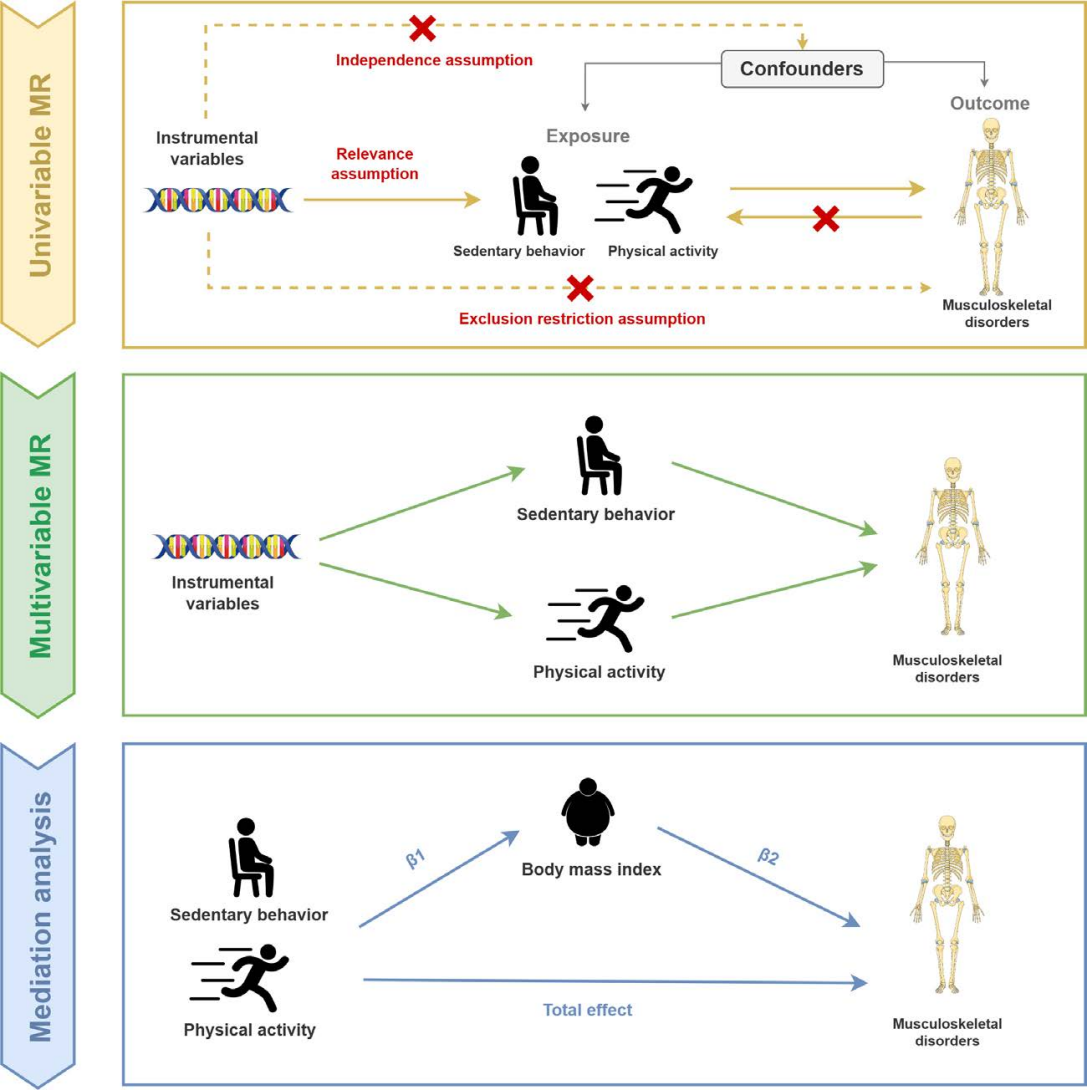
Research flowchart.

In this study, we first performed 2-sample univariable MR (UVMR) analysis with SB or PA as exposure and 13 MSDs as outcomes, respectively. Reverse MR analysis and genetic correlation analysis were then used to verify the directionality and genetic correlations. Additionally, MVMR analysis and mediation analysis were conducted to assess the independent causal effects and to explore the potential mechanisms. The research flowchart is presented in Figure 1. MR design must be based on 3 basic assumptions: IVs are strongly associated with the exposure; IVs are independent of potential confounders; and IVs affect the outcome only through the exposure.

Our study followed the STROBE guidelines. Our MR study was a re-analysis of publicly available genome-wide association studies (GWAS) data, so no additional ethical approval was required for our study.

### 2.2. Data sources

The genetic instruments for SB and PA were sourced from the GWAS Catalog (https://www.ebi.ac.uk/gwas/). We used self-reported leisure screen time (LST) as an indicator of SB. The summary statistics for LST (including time spent watching TV, playing videogames, etc) were derived from a GWAS meta-analysis of 51 studies, incorporating 526,725 samples of European ancestry.^[22]^ Regarding the PA phenotype, we used the self-reported moderate-to-vigorous intensity physical activity (MVPA) data from a GWAS analysis by Klimentidis et al,^[27]^ which were measured by a touchscreen questionnaire in a manner similar to the International Physical Activity Questionnaire.^[28]^ MVPA was defined as the total number of minutes per week of moderate physical activity multiplied by 4, and the total number of minutes per week of vigorous physical activity multiplied by 8, corresponding to their metabolic equivalents.

Summary-level data for 13 MSDs were all retrieved from the FinnGen consortium. These MSDs can be categorized as follows: spinal disorders (scoliosis, spinal stenosis, spondylolisthesis, low back pain, and cervicobrachial syndrome); bone disorders (osteoporosis, osteonecrosis, and osteomyelitis); soft tissue disorders (rotator cuff syndrome, meniscus derangement, and lateral epicondylitis); and inflammatory joint disorders (rheumatoid arthritis and gout).

Genetic variants associated with BMI were obtained from the Genetic Investigation of ANthropometric Traits consortium.^[29]^

Details of these GWAS data is summarized in Table S1 (Supplemental Digital Content, https://links.lww.com/MD/Q25).

### 2.3. Selection of instrumental variables

Significant and independent SNPs of LST and MVPA were selected as IVs for subsequent analysis according to the following criteria. We first selected SNPs at the genome-wide significance threshold (*P* < 5 × 10^−8^). These SNPs were then clumped for linkage disequilibrium (LD) (*r*^2^ = 0.001, kb = 10,000). And SNPs with *F* statistics <10 were removed. Additionally, a harmonization process was undertaken, and ambiguous and palindromic SNPs were removed.

### 2.4. Statistical analysis

The inverse-variance weighted (IVW) method was used as the primary method for MR causal estimates, while MR-Egger regression and weighted median were used as complements. Consistent estimate directions across these 3 methods can enhance the robustness of our results.

We performed UVMR analysis to evaluate the causal effects of LST/MVPA on the risk of 13 MSDs. Subsequently, we conducted reverse MR analysis. The analytical methods and process were consistent with those in the preceding analysis.

The linkage disequilibrium score regression (LDSC) was applied to estimate the genetic correlations (*r*_*g*_) between LST/MVPA and MSDs from GWAS summary data. This method can assess the association between test statistics and LD to quantify the contribution of inflation from true polygenic signal or bias.^[30]^

Considering the strong genetic correlation between LST and MVPA, we performed MVMR analysis for LST and MVPA with adjustment for one another to estimate their independent causal effects on MSDs.^[22]^

Furthermore, to elucidate the potential pathway by which LST/MVPA affected MSDs, we conducted a 2-step mediation analysis to assess the potential mediating role of BMI in the identified associations. In the first step, we obtained the causal effect (β1) of the exposure on the mediator. The second step was to estimate the effect (β2) of the mediator on MSDs. Using the product of coefficients method, we multiplied these 2 estimates together as the indirect effect (β1 × β2), that is, mediating effect. The total effect consisted of direct effect (the effect of the exposure on MSDs independent of the mediator) and indirect effect (the effect of the exposure on MSDs via the mediator). The proportion mediated by the mediator was calculated by dividing the mediating effect by the total effect.

A series of sensitivity analyses were applied to assess the robustness of the MR results. We employed Cochran *Q* test to assess heterogeneity among SNP, with *P* < .05 signifying the presence of heterogeneity. The MR-Egger intercept test was used to identify potential horizontal pleiotropy. Furthermore, MR pleiotropy residual sum and outlier (MR-PRESSO) method was employed to detect significant outliers.

We used the “TwoSampleMR” (version 0.5.6) package in R (version 4.3.2) to perform all the MR analysis. MR estimates were expressed as odds ratios with 95% confidence intervals (CIs). To correct for multiple testing, we applied Benjamini–Hochberg method to control the false discovery rate (FDR). FDR-adjusted *P* < .05 was considered statistically significant.

## 3. Results

### 3.1. Causal effects of LST on MSDs

After strict quality control criteria, 117 and 19 SNPs were identified as IVs for LST and MVPA, respectively. The *F* statistics were all >10, indicating no weak IVs (Table S2, Supplemental Digital Content, https://links.lww.com/MD/Q25).

**Figure 2.**
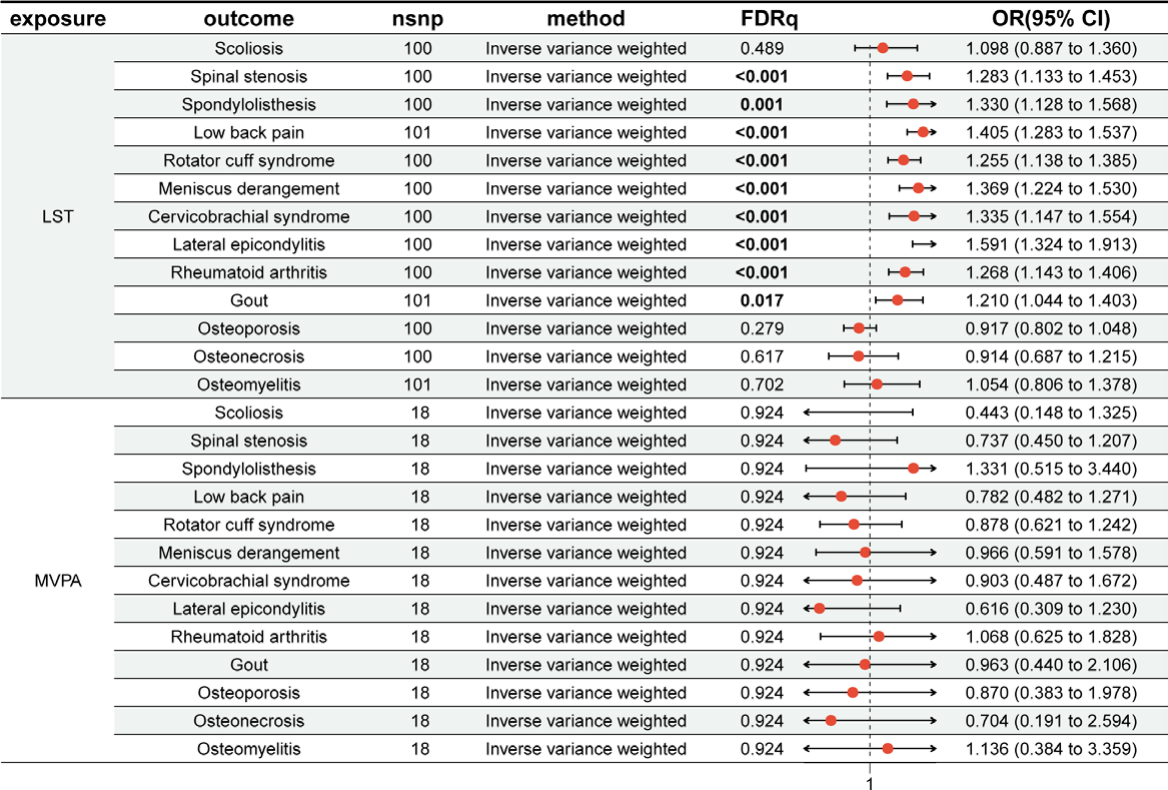
Univariable Mendelian randomization estimates for the causal effects of LST and MVPA on MSDs. After Benjamini–Hochberg correction, LST was significantly associated with an increased risk of 8 MSDs; MVPA showed no causal effect on MSDs. OR and 95% CI were calculated using the inverse-variance weighted method. FDR-adjusted *P* < .05 was considered statistically significant. CI = confidence interval, LST = leisure screen time, MSDs = musculoskeletal disorders, MVPA = moderate-to-vigorous intensity physical activity, nSNP = number of single nucleotide polymorphisms, OR = odds ratio.

After Benjamini–Hochberg correction, IVW method suggested that genetically predicted LST was significantly associated with an increased risk of 8 MSDs, including spinal stenosis (OR = 1.283, 95% CI: 1.133–1.453; *P* = .0002), spondylolisthesis (OR = 1.330, 95% CI: 1.128–1.568; *P* = .0012), low back pain (OR = 1.405, 95% CI: 1.283–1.537; *P* = 2.4E−12), cervicobrachial syndrome (OR = 1.335, 95% CI: 1.147–1.554; *P* = .0004), rotator cuff syndrome (OR = 1.255, 95% CI: 1.138–1.385; *P* = 1.8E-5), meniscus derangement (OR = 1.369, 95% CI: 1.224–1.530; *P* = 1.7E−7), lateral epicondylitis (OR = 1.591, 95% CI: 1.324–1.913; *P* = 2.8E−6), and gout (OR = 1.210, 95% CI: 1.044–1.403; *P* = .0174) (Fig. 2; Table S3, Supplemental Digital Content, https://links.lww.com/MD/Q25). All 3 methods yielded consistent effect directions, supporting the robustness of the findings. IVW method identified a significant association between LST and rheumatoid arthritis, whereas MR-Egger method showed directional inconsistency.

Cochran *Q* test detected heterogeneity in SNP estimates (*P* < .05), but it was acceptable since we used the random-effect IVW for MR estimates (Table S5, Supplemental Digital Content, https://links.lww.com/MD/Q25). MR-Egger intercept tests indicated no presence of horizontal pleiotropy. MR-PRESSO detected several outliers for several MSDs; however, these associations persisted after removing outliers (Table S6, Supplemental Digital Content, https://links.lww.com/MD/Q25).

### 3.2. Causal effects of MVPA on MSDs

In UVMR analysis, IVW method indicated no causal association between genetically predicted MVPA and 13 MSDs after Benjamini–Hochberg correction. And the other methods showed similar results (Fig. 2; Table S4, Supplemental Digital Content, https://links.lww.com/MD/Q25).

Heterogeneity was detected in the Cochran *Q* test. MR-Egger intercept tests indicated potential horizontal pleiotropy in the analysis for spinal stenosis and gout. MR-PRESSO detected outliers in the associations with several MSDs (Table S5, Supplemental Digital Content, https://links.lww.com/MD/Q25). After removing the outliers, the re-analysis results showed no pleiotropy (Table S6, Supplemental Digital Content, https://links.lww.com/MD/Q25).

### 3.3. Reverse MR and genetic correlation analysis

In the reverse MR analysis, we identified SNPs significantly and independently associated with each MSD. In case of few SNP identified, a less strict threshold (*P* < 5 × 10^−6^) was applied. IVW results suggested that low back pain was positively associated with LST (OR = 1.145, 95% CI: 1.041–1.260; *P* = .005), while spinal stenosis was negatively associated with MVPA (OR = 0.982, 95% CI: 0.967–0.997; *P* = .021) (Table S7, Supplemental Digital Content, https://links.lww.com/MD/Q25).

Based on the results of UVMR analysis, LDSC analysis was conducted for the identified associations. The results supported that all 8 identified MSDs had significant positive genetic correlations with LST (Table S8, Supplemental Digital Content, https://links.lww.com/MD/Q25).

### 3.4. MVMR analysis and mediation analysis

We further performed MVMR to evaluate the independent effects of LST after adjustment for MVPA, and vice versa. IVs used in MVMR are shown in Table S9, Supplemental Digital Content, https://links.lww.com/MD/Q25.

**Figure 3.**
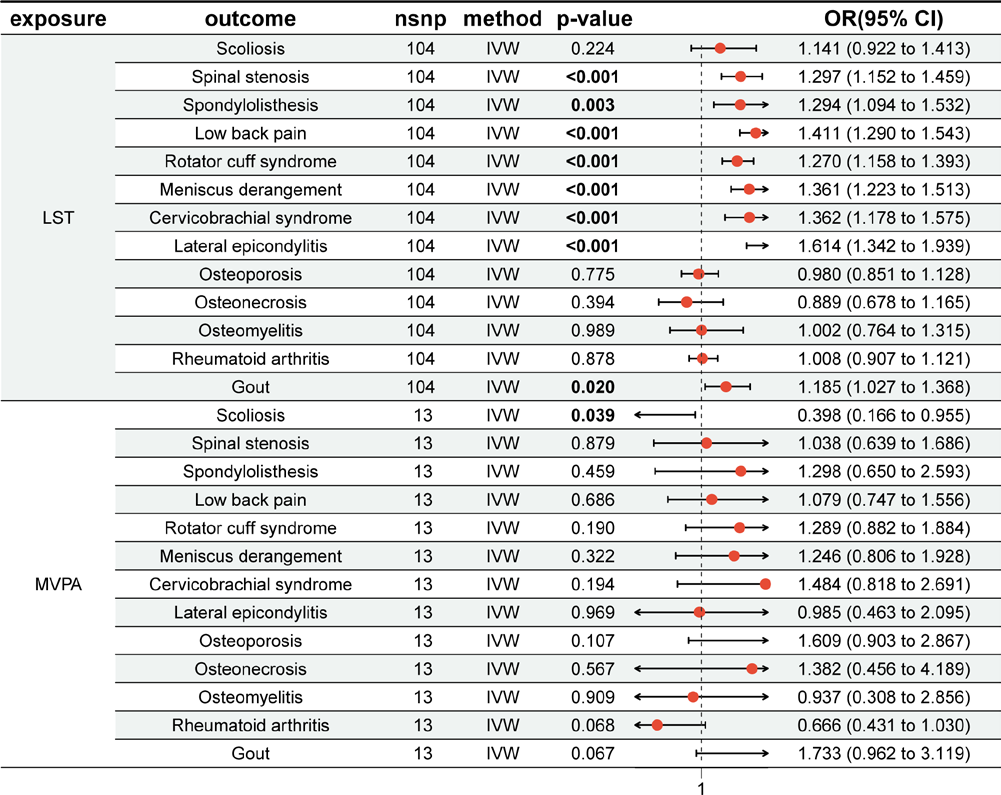
Multivariable Mendelian randomization estimates for the independent effects of LST and MVPA on MSDs. LST was still significantly associated with 8 MSDs after adjusting for MVPA; MVPA became significantly associated with scoliosis after adjusting for LST. OR and 95% CI were calculated using the inverse-variance weighted method. FDR-adjusted *P* < .05 was considered statistically significant. CI = confidence interval, IVW = inverse-variance weighted, LST = leisure screen time, MSDs = musculoskeletal disorders, MVPA = moderate-to-vigorous intensity physical activity, nSNP = number of single nucleotide polymorphisms, OR = odds ratio.

After adjusting for MVPA, the associations between LST and all the 8 MSDs remained significant. After adjustment for LST, the associations between MVPA and scoliosis became significant (OR = 0.398, 95% CI: 0.166–0.955; *P* = .0391), while the other associations remained non-significant (Fig. 3; Table S10, Supplemental Digital Content, https://links.lww.com/MD/Q25).

Additionally, BMI mediated 9.72%–65.1% of the causal effects of LST on 8 MSDs (Table 1; Table S11, Supplemental Digital Content, https://links.lww.com/MD/Q25).

**Table 1:** Proportion mediated by BMI in the associations between LST and 8 MSDs, based on 2-step Mendelian randomization mediation analysis.

Exposure	Mediator	Outcome	Proportion mediated (%)
LST	BMI	Spinal stenosis	40.4
LST	BMI	Spondylolisthesis	31.9
LST	BMI	Low back pain	20.6
LST	BMI	Rotator cuff syndrome	22
LST	BMI	Meniscus derangement	30.1
LST	BMI	Cervicobrachial syndrome	17.1
LST	BMI	Lateral epicondylitis	9.72
LST	BMI	Gout	65.1

The indirect (mediated) effect was estimated using the product of coefficients method. The proportion mediated was calculated by dividing the indirect effect by the total effect.

BMI = body mass index, LST = leisure screen time, MSDs = musculoskeletal disorders.

## 4. Discussion

The current MR study first comprehensively investigated the causal associations between LST/MVPA and MSDs using multiple MR approaches. The results provided robust evidence that LST was a risk causal factor for 8 MSDs independent of MVPA. UVMR found no causal effect of MVPA on the risk of MSDs; however, MVMR identified the causal effect of MVPA on scoliosis. BMI was a potential mediator in the causal associations between LST and MSDs.

Previous observational studies have noted the associations between sedentary behavior and spinal disorders. A cross-sectional population study suggested that sedentary work may increase the risk of developing spondylolisthesis.^[31]^ A 2-sample MR study identified the causal associations between duration of mobile phone use and time spent watching TV with the risk of spinal stenosis.^[32]^ Meanwhile, individuals with spinal stenosis have been observed to be highly sedentary and less likely to meet physical activity guidelines of 150 minutes of MVPA per week, which was consistent our reverse analysis findings.^[33]^ Additionally, objectively measured leisure-time sitting was found to be positively associated with low back pain intensity,^[34]^ a finding supported by several MR analyses.^[19,20,35]^ Numerous studies have also supported the association between SB and low back pain, as well as pain in the arm, neck, and/or shoulder.^[8,36–39]^ In line with these findings, our study revealed the causal associations between SB and the risk of spondylolisthesis and cervicobrachial syndrome, as well as the bidirectional causal association between SB and low back pain. These findings provided genetic evidence for the causal nature of these associations, which further confirmed and extended the findings from previous observational and MR studies. Notably, while reverse MR analysis suggested potential reverse associations, such findings should be interpreted with caution. Reverse MR does not imply temporal causality in the traditional sense, but rather reflects lifetime genetic predisposition. The bidirectionality with low back pain may reflect a behavioral feedback loop in which prolonged sedentary time increases the risk of pain, while individuals with pain may also adopt more sedentary lifestyles as a coping mechanism. This underscores the complex interplay between symptoms and behaviors, which should be interpreted in the context of underlying behavioral and clinical dynamics. Further longitudinal investigation is warranted to verify the directionality observed in reverse MR analysis.

Rotator cuff syndrome, lateral epicondylitis, and meniscus derangement are typically associated with forceful repetitive use or overuse of periarticular soft tissues. However, these conditions are also seen in sedentary populations. For example, while rotator cuff disorder commonly occurs in physically demanding occupations, it is noteworthy that these conditions also affect sedentary individuals.^[40,41]^ A prospective cohort study suggested that knees abnormalities, including meniscal degeneration and tears, were highly prevalent in asymptomatic sedentary individuals.^[42]^ Additionally, a sedentary lifestyle was found to be implicated in the pathology of tendon injuries.^[43,44]^ However, despite these findings, there is currently a lack of direct evidence linking SB to these diseases. Our MR study filled the gap and provided novel evidence on the associations between SB and the risk of soft tissue disorders which warrant further investigation.

The associations between SB and arthritis, especially rheumatoid arthritis (RA), have been extensively documented, with most studies focusing on RA. Our study first identified the causal association of SB with gout, which confirmed the findings from a prospective cohort study that individuals reporting longer sedentary time had higher risk of gout.^[11]^

Regarding osteoporosis, some studies noted a negative association between sedentary time and bone mineral density, a diagnostic index of osteoporosis^[45,46]^; however, there are conflicting conclusions from observational studies.^[47,48]^ Our study found no association between SB and osteoporosis, osteonecrosis, osteomyelitis, which was supported by another MR study.^[49]^ The inconsistent results may be attributed to the influence of confounding factors, such as smoking.

Notably, while MVPA did not show causal associations with MSDs in the UVMR analysis, the MVMR revealed a significant protective effect of MVPA on scoliosis after adjusting for LST. This finding is biologically plausible, and may reflect a statistical suppression effect. MVPA and LST are genetically and behaviorally highly correlated. Adjusting for LST allows the independent role of MVPA to emerge by accounting for the confounding or suppressing effect of sedentary behavior. Mechanistically, scoliosis – especially in its adolescent idiopathic form – has been linked to poor postural control, poor trunk muscle control, and spinal muscle imbalance, while physical activity may help correct or mitigate these conditions through improved posture regulation, neuromuscular coordination, spinal stabilization, and musculoskeletal development. It is possible that the beneficial impact of MVPA on scoliosis risk was previously obscured by the dominant adverse effects of LST. This finding underscores the importance of considering interdependent behavioral exposures and their confounding effects in causal inference models. Consistent with our findings, a meta-analysis found that moderate and vigorous PA reduced the odds of being diagnosed with adolescent idiopathic scoliosis by 13% and 24%, respectively.^[50]^ These benefits may stem from PA’s role in enhancing muscle strength and bone density, suggesting its potential as an effective prevention strategy.^[51,52]^

Several recent MR investigations on SB and MSDs largely support our findings but also reveal important nuances. Zhao et al and Qiu et al reported positive associations between screen-based sedentary time and low back pain or intervertebral disc disorders, while Cao et al and Huang et al reported associations with RA.^[19–21,53]^ Compared to these focused analyses, our study examined a broader spectrum of 13 MSDs and confirmed causal effects of LST on 8 of them. Regarding PA, recent MR studies have yielded inconsistent findings: some found no protective effect of MVPA, while others noted differential effects depending on intensity or total activity.^[54–56]^ Our multivariable MR revealed a protective association between MVPA and scoliosis after adjusting for LST. These discrepancies may stem from differences in phenotype definitions, measurement methods (e.g. self-reported vs accelerometer-based), and analytic approaches.

A common misconception is that SB is merely a lack of physical activity.^[57]^ More accurately, the latter should be referred to as physical inactivity, which is a separate construct from SB.^[58]^ There is a growing body of evidence that SB has emerge as a distinct public health risk independent of PA. SB has been shown to be associated with exacerbated systemic inflammation regardless of the anti-inflammatory effect of PA.^[16]^ Even when adults meet the recommendations for PA, their health can still be compromised by prolonged sedentary time.^[58]^ Our MR study further supported these observational results by revealing the independent causal effect of SB on musculoskeletal health.

Mechanisms underlying the associations between SB and MSDs are complex and multifaceted. Obesity appears to be one potential mediator involved in this pathway. Our findings align with previous studies showing that BMI may partially mediate the SB-MSDs pathway. The association of SB with BMI has been well described,^[59–62]^ while a high BMI is associated with multiple musculoskeletal symptoms, including spinal stenosis, spondylolisthesis, rotator cuff tendonitis, lateral epicondylitis, tendinopathy, meniscus derangement, intervertebral disc degeneration, low back pain, pain in the neck/shoulders, and gout.^[32,63–71]^ Biomechanics offers a partial explanation for the effect of BMI on MSDs: obesity may predispose to the dysfunction, degeneration, or injuries of the spine, joints, and soft tissues by inducing postural deviation, increased mechanical stress, muscle fatigue, and deleterious biomechanical effects.^[72–77]^ Spinal curvature – particularly in the lumbar region – has been shown to be critical for spinal mechanical stability, which may be impaired by prolonged sedentary postures and thereby contributes to the development of MSDs.^[78]^ Additionally, obesity-related metabolic factors may be another crucial part of the underlying mechanisms. Obesity is accompanied by metabolic dysfunction and chronic low-grade inflammation, both of which are implicated in the pathogenesis of MSDs.^[23,79–81]^ Of note, MSDs at different sites of the body vary in pathology, risk factors, and biological mechanisms. Consequently, more in-depth studies are necessitated to identify other potential mediators and to explore more specific mechanisms.

The primary strength of our study is the MR design, which could reduce potential reverse causality and confounding bias that exist in observational studies, thereby enhancing causal inference. Second, the population was restricted to European ancestry to reduce potential bias introduced by population stratification. Additionally, we further applied MVMR and mediation analysis to minimize the pleiotropic effects and reveal the potential mechanisms. More importantly, our study complemented the current evidence on the effect of SB on MSDs and further corroborated the causality.

Nonetheless, several limitations need to be acknowledged. Firstly, our results were mainly based on European population, which may not apply to other ethnic groups. Secondly, the use of summary-level data precluded us from conducting subgroup analysis. Thirdly, despite the use of various analytical methods and sensitivity tests, it could not be guaranteed that all possible pleiotropic effects had been completely eliminated, so the results needed a cautious overall interpretation. Additionally, our study relied on self-reported data of SB and PA rather than objective measures, which may be subject to recall bias. Also of note, as suggested by several MR analyses, different phenotypes of SB (such as TV watching and computer use) may exert different effects on MSDs, implying a biological heterogeneity underlying different SB phenotypes.^[19,35,53]^ Meanwhile, different patterns of sedentary time accumulation can also make a difference, even with the same total sedentary time.^[82]^ Therefore, using LST as a proxy for SB may neglect the internal complexity and diversity in SB. For a better understanding of the impact of SB, future studies should take into account the frequency, interruption, duration, and types of SB.

## 5. Conclusions

In conclusion, our study provides genetic evidence supporting the causal role of SB in MSDs, and highlights the mediating role of BMI. However, PA has little protective effect on MSDs. These insights contribute to the existing body of knowledge and offer valuable references for the development of evidence-based prevention and interventions for MSDs.

## Acknowledgements

We thank the participants and the researchers of the genome-wide association studies who made their summary statistics publicly available for this study.

## Author contributions

**Conceptualization:** Xiaoyan Zhang, Yuqiang Li.

**Data curation:** Xiaoyan Zhang, Yuqiang Li.

**Formal analysis:** Xiaoyan Zhang, Yuqiang Li.

**Investigation:** Xiaoyan Zhang, Yuqiang Li.

**Methodology:** Xiaoyan Zhang, Yuqiang Li.

**Software:** Xiaoyan Zhang.

**Writing – original draft:** Xiaoyan Zhang.

**Writing – review & editing:** Yuqiang Li.

## Supplementary Material


